# Diagnosis and Interim Treatment Outcomes from the First Cohort of Multidrug-Resistant Tuberculosis Patients in Tanzania

**DOI:** 10.1371/journal.pone.0062034

**Published:** 2013-05-13

**Authors:** Stellah G. Mpagama, Scott K. Heysell, Nora D. Ndusilo, Happiness H. Kumburu, Isack A. Lekule, Riziki M. Kisonga, Jean Gratz, Martin J. Boeree, Eric R. Houpt, Gibson S. Kibiki

**Affiliations:** 1 Kibong’oto National Tuberculosis Hospital, Kilimanjaro, Tanzania; 2 Kilimanjaro Clinical Research Institute, Kilimanjaro Christian Medical Centre, Moshi, Tanzania; 3 University of Virginia, Charlottesville, Virginia, United States of America; 4 UMC St Radboud/UCCZ Dekkerswald, Nijmegen, The Netherlands; London School of Hygiene and Tropical Medicine, United Kingdom

## Abstract

**Setting:**

Kibong’oto National Tuberculosis Hospital (KNTH), Kilimanjaro, Tanzania.

**Objective:**

Characterize the diagnostic process and interim treatment outcomes from patients treated for multidrug-resistant tuberculosis (MDR-TB) in Tanzania.

**Design:**

A retrospective cohort study was performed among all patients treated at KNTH for pulmonary MDR-TB between November 2009 and September 2011.

**Results:**

Sixty-one culture-positive MDR-TB patients initiated therapy, 60 (98%) with a prior history of TB treatment. Forty-one (67%) were male and 9 (14%) were HIV infected with a mean CD4 count of 424 (±106) cells/µl. The median time from specimen collection to MDR-TB diagnosis and from diagnosis to initiation of MDR-TB treatment was 138 days (IQR 101–159) and 131 days (IQR 32–233), respectively. Following treatment initiation four (7%) patients died (all HIV negative), 3 (5%) defaulted, and the remaining 54 (89%) completed the intensive phase. Most adverse drug reactions were mild to moderate and did not require discontinuation of treatment. Median time to culture conversion was 2 months (IQR 1–3) and did not vary by HIV status. In 28 isolates available for additional second-line drug susceptibility testing, fluoroquinolone, aminoglycoside and para-aminosalicylic acid resistance was rare yet ethionamide resistance was present in 9 (32%).

**Conclusion:**

The majority of MDR-TB patients from this cohort had survived a prolonged referral process, had multiple episodes of prior TB treatment, but did not have advanced AIDS and converted to culture negative early while completing an intensive inpatient regimen without serious adverse event. Further study is required to determine the clinical impact of second-line drug susceptibility testing and the feasibility of alternatives to prolonged hospitalization.

## Background

Multidrug-resistant tuberculosis (MDR-TB), defined as resistance to both isoniazid and rifampin, remains poorly diagnosed and treated [1]. Of the 440,000 estimated incident cases annually in 2008, only 7% were reported to the World Health Organization (WHO) [2] and of those, only a fifth were treated according to recommended guidelines [1]. Treatment outcomes are inferior to drug-susceptible TB in part because medications used in the treatment of MDR-TB are less potent and associated with a greater number of side effects, and duration is recommended to last at least 20 months which can compromise adherence [3], [4]. The second-line drugs used in the treatment of MDR-TB have only recently been available in many TB endemic countries following WHO rollout initiatives. Analysis of outcomes from diverse settings with recent access to MDR-TB treatment is therefore critical for study of comparative efficacy and for design of similar programs in other emerging locations.

Tanzania is a WHO designated high burden TB country [2]. Although data on drug-resistance are limited, a recent cluster survey placed the prevalence from MDR-TB among retreatment cases at only 3.9% [5]. Treatment for MDR-TB was made available in 2009 and the Ministry of Health selected Kibong’oto National Tuberculosis Hospital (KNTH) in the Kilimanjaro region of Northern Tanzania as the initial referral hospital for all MDR-TB cases [6]. Treatment consisted of a standardized MDR-TB regimen based only on drug susceptibility results for the first-line medications isoniazid, rifampin, streptomycin and ethambutol. Therefore, MDR-TB in Tanzania reflects a unique population with little to no exposure to second-line medications and with a relatively low background prevalence of drug resistance that may lend to favorable outcomes if patients are treated with a standardized MDR-TB regimen.

However, given that long-term outcomes such as relapse following treatment of MDR-TB can take years to determine, correlative intermediate markers have been used. The most studied marker in pulmonary TB has been sputum culture conversion from positive to negative [7]–[9] and MDR-TB patients that fail to convert their sputum by two months are less likely to achieve cure [10]. Therefore, the following study describes the diagnostic referral process, clinical presentation and interim treatment outcomes including the duration of sputum culture conversion from the first cohort of patients treated for MDR-TB in Tanzania. Furthermore, among the available pretreatment *M. tuberculosis* isolates, drug-susceptibility testing was performed to determine the range of resistance to drugs within the standardized regimen that may additionally predict outcome or inform further programmatic decisions.

## Methods

### Study Design

KNTH hospital staff reviewed consecutive medical charts from patients referred for MDR-TB treatment from November 2009 to September 2011. Patients were considered eligible for inclusion if they had a *Mycobacterium tuberculosis* isolate with resistance to isoniazid and rifampin and had been started on a MDR-TB regimen. The institutional review board at Kilimanjaro Christian Medical Center (KCMC) and the University of Virginia approved this study.

### Study Site

KNTH was a former sanatorium, and is currently the country’s only referral hospital for MDR-TB management with a dedicated 40-bed capacity in addition to separate facilities for treatment of drug-susceptible TB. Forty kilometers from KNTH, the biotechnology laboratory affiliated with the Kilimanjaro Clinical Research Institute performs smear microscopy, mycobacterial culture and first-line susceptibility testing by Bactec MGIT (BD, Franklin Lakes, USA), and DNA probe for *Mycobacterium tuberculosis* complex (Gen-Probe, San Diego, USA). The laboratory has completed external proficiency testing. Second-line medication minimum inhibitory concentrations (MICs) on MYCOTB Sensititre plates (TREK Diagnostics, Cleveland, USA) have only recently been available and as such, the MICs reported were performed on archived pretreatment isolates and not available for therapeutic decisions in this initial cohort. Repeat DNA probe and sequencing of non-tuberculous mycobacteria was later performed at the University of Virginia. For study inclusion, only the initial susceptibility performed at the national TB reference laboratory in Dar es Salaam was utilized.

### MDR-TB Referral and Treatment Program

A MDR-TB suspect’s specimens were transported to the national TB reference laboratory based on a series of mechanisms including routine clinical practice/physician suspicion, drug-resistance surveillance, and independent research [11]. The national TB reference laboratory performed mycobacterial culture, biochemical speciation and first-line susceptibility testing by proportion method on Lowenstein-Jensen slants.

Culture positive MDR-TB patients were treated at KNTH with a standardized regimen during the inpatient intensive phase with one injectable agent (kanamycin or pending stock availability, amikacin or capreomycin), a fluoroquinolone (levofloxacin or pending stock availability, ofloxacin), ethambutol (if susceptible), pyrazinamide, ethionamide and cycloserine. Drugs were directly administered seven days per week and dosed by weight [11]. In case of severe adverse event, the dosage was modified or the drug substituted. Para-aminosalicylic acid (PAS) was available for those requiring substitution. The inpatient regimen was continued for at least 4 months following culture conversion, defined as two consecutive negative cultures at least one month apart, and a minimum of 6 months. Then if symptomatically improved, subjects were eligible for discharge to complete an additional 18 months of a continuation phase that did not include the injectable agent. Every patient was tested for HIV and antiretroviral therapy (ART) was initiated, if indicated, within 8 weeks of MDR-TB treatment initiation [11]. ART regimens were either (1) stavudine or zidovudine plus lamivudine plus efavirenz or nevirapine, or (2) tenofovir plus emtricitabine plus efavirenz.

Prior to treatment initiation, sputum was collected for smear microscopy and culture on Lowenstein-Jensen solid agar, and monthly thereafter until the end of intensive phase. Clinical examination was performed daily and laboratory parameters including hematology and a comprehensive metabolic panel were monitored monthly. Chest radiographs were taken prior to treatment initiation and at 6 months per KNTH protocol.

### Definitions and Outcome Measures

For purposes of analysis, adverse events were categorized as mild if an asymptomatic laboratory parameter change necessitated clinical observation only; moderate if minimal or non-invasive intervention was indicated but without alteration to the MDR-TB drug regimen; or severe if medically significant and treatment alteration was necessary [12]. For comparison of pretreatment and 6 month chest radiographs, the extent of affected lung was determined by zonal score and reported as percentage of the total lung [13]. Weight (kg) was recorded pretreatment and at 6 months. Treatment outcomes were determined following the inpatient phase of therapy, and death was categorized as occurring by any cause; default, if treatment was interrupted for greater than one week; and intensive phase complete; if the injectable agent was discontinued and the patient discharged on an oral continuation phase regimen. KNTH staff traced defaulters and if treatment was resumed with <2 months interruption, then the subject was eligible for inclusion in analysis of intensive phase completion.

All statistical tests were two-tailed with a p-value <0.05 considered significant. Results were expressed as simple proportions with mean (95% Confidence Interval) or median (interquartile range) when appropriate. Means were compared by paired t-test and medians with the Mann Whitney U test for non-parametric data.

## Results

### Demographics and Clinical Characteristics

**Figure 1:**
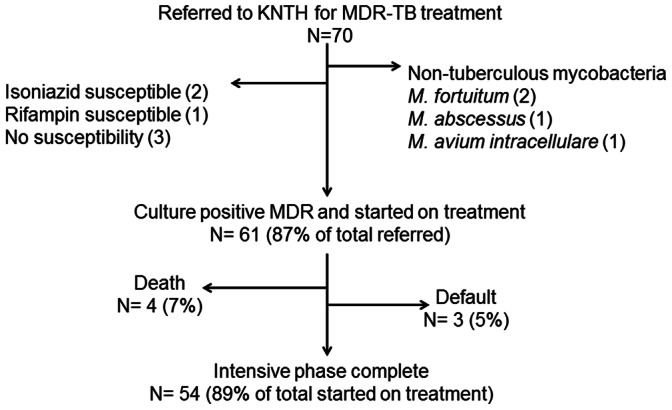
Patients referred to KNTH for MDR-TB treatment. KNTH =  Kibong’oto National TB Hospital, Tanzania. MDR-TB =  multidrug-resistant tuberculosis. All patients had pulmonary TB.

A total of 70 patients were referred to KNTH from November 2009 through September 2011 for MDR-TB treatment (Figure 1). Sixty-one (87%) patients were referred based on routine clinical practice/physician suspicion, while the remainder by drug-resistance surveillance or independent research, including 8 (12%) for whom initial screening susceptibility was made by molecular detection of drug-resistant mutations for isoniazid and rifampin (MTBDR*plus,* Hain Lifescience, Nehren, Germany). For two patients initial drug susceptibility testing results could not be confirmed and another 3 patients were found to have a *M. tuberculosis* isolate which was sensitive to isoniazid or rifampin, and thus excluded from the analysis. Four patients were further excluded as their isolate was later identified as non-tuberculous mycobacteria (NTM) including two patients with *M. fortuitum*, and one each with *M. abscessus* and *M. avium* complex (Figure 1).

Thus, 61 patients were included in the analysis and all had pulmonary TB with only one additionally having an extrapulmonary focus. Forty-two (69%) were from Dar es Salaam but the remainder of patients represented diverse geographic locations in the country (Table 1). The average age was 36 years (±13), and 41 (67%) were male. Nine (14%) had HIV with a mean CD4+ lymphocyte count of 424 cells/µl (range 317 - 530). Six (67%) of the nine patients with HIV were on ART prior to MDR-TB treatment. Diabetes mellitus was rare and found in only 2 (3%).

**Table 1:** Baseline characteristics for those initiating MDR-TB treatment (N = 61).

Characteristic	Subcategory	Number
Age, mean years (±SD)	N/A	36 (13)
Gender (%N)	Male	41 (67)
	Female	20 (33)
Cigarette smoking (%N)	Yes	16 (26)
	No	21 (34)
	Unknown	24 (39)
Alcohol use (%N)	Yes	9 (15)
	No	29 (47)
	Unknown	23 (38)
Region of referral/domicile (%N)	Dar es Salaam	42 (69)
	Arusha	4 (7)
	Tanga	4 (7)
	Kilimanjaro	2 (3)
	Zanzibar	2 (3)
	Others	7 (11)
History of previous TB (%N)	Yes	60 (98)
	No	1 (2)
Number of prior TB episodes (% with previous TB)	One	8 (13)
	Two	29 (48)
	Three	12 (20)
	Four or more	11 (18)
Outcome of last TB episode (% with previous TB)	Favorable	15 (25)
	Unfavorable	45 (75)
Time from specimen collection to MDR-TB diagnosis, median days (IQR)	N/A	138 (101–159)
HIV status (%N)	Positive*	9 (15)
	Negative	52 (85)
CD4 cells/µl, mean (±SD) among HIV positive	N/A	424 (106)
Diabetes (%N)	Yes	2 (3)
	No	59 (97)
Cavitary disease†	Yes	22 (46)
	No	26 (54)

N/A =  not applicable.

* 6 (67%) of HIV positive patients were on antiretroviral therapy prior to referral for MDR-TB treatment.

† Forty-eight baseline chest radiographs were available for analysis.

Sixty patients (98%) had a prior history of TB of which 52 (87%) had two or more episodes of the infection in the past. Forty-five (75%) had an unfavorable outcome during the last episode that resulted in drug-susceptibility testing and the diagnosis of MDR-TB. The median time between sputum specimen collection and diagnosis of MDR-TB was 138 days (IQR 101–159). The median time from diagnosis of MDR-TB to initiation of MDR-TB treatment regimen was 131 days (IQR 32–233).

### Treatment Regimens and Adverse Events

**Figure 2:**
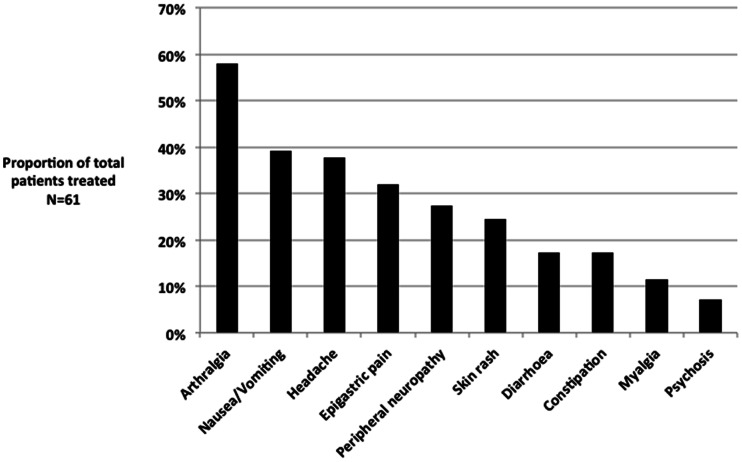
Prevalence of medication related symptoms. The prevalence of medication related symptoms during the inpatient intensive phase occurring at least once in an individual patient, as determined by percentage of total patients (N = 61).

All patients received the standardized MDR-TB treatment regimen. Ethambutol resistance was present in 43 (71%). Twenty-one (34%) of patients had a medication changed during treatment; 17 changes (81%) were due to stock shortage, while 4 (19%) were because of a severe adverse event (all with nephrotoxicity). Liver function remained within acceptable range for all patients and no serious hepatoxicity was noted. Three patients developed psychosis that resolved following temporary discontinuation of cycloserine and treatment with antipsychotic agents. Of the 61 patients, 49 (80%) experienced mild or moderate adverse events potentially related to medication (Figure 2). Of the 9 HIV infected patients, only 1 (11%) developed a severe adverse event (nephrotoxicity), but the remaining 8 had at least one mild or moderate adverse event.

### Interim Treatment Outcomes

**Figure 3:**
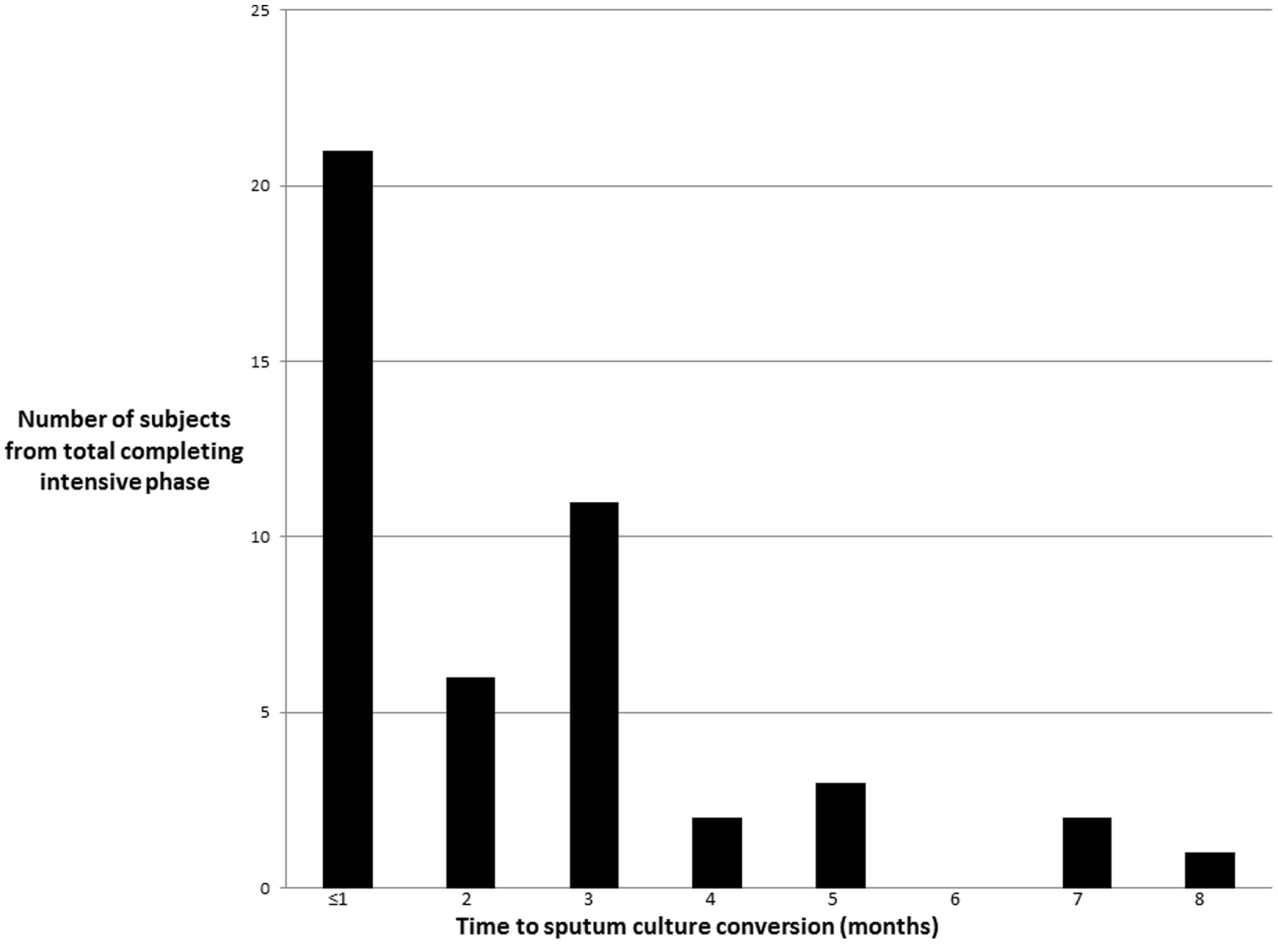
Distribution of the time to sputum culture conversion. Legend: Sputum was collected for culture prior to treatment initiation and monthly thereafter. Culture conversion was defined as the first of two consecutive months of negative cultures. Eight subjects were excluded as regular follow-up culture results were unavailable.

Fifty-four (89%) patients completed the intensive phase and were discharged on an oral continuation regimen (Table 2). The median duration of the intensive phase was 7 months (IQR 6–8). Among HIV infected patients, 8 (89%) completed the intensive phase which did not differ from the HIV uninfected patients. A total of 4 patients (8%) died, all during the first month of treatment, and none had HIV (Table 2). An additional three patients (5%) defaulted and upon tracing one had died, one was restarted on MDR-TB therapy following 3 months of interruption, and the other was lost to follow-up. With regard to the time required for sputum culture conversion, 8 (13%) patients had missing data and could not be included. Thus of the remaining 46 (85%), the median time to sputum culture conversion was 2 months (IQR 1–3) and did not differ by HIV status (p = 0.8). Nevertheless, 18 (39%) patients required ≥3 months for sputum culture conversion, with a maximum of 8 months (Figure 3).

**Table 2:** Comparison of patients that completed intensive phase of MDR-TB therapy to those that died.

Characteristic	Intensive phase complete, N = 54*	Died, N = 4	p-value
Age, mean years (SD)	35 (13)	31(13)	0.43
Gender, male (%N)	36 (67)	3(75)	0.6
HIV infected (%N)	8 (15)	0	0.4
Mean number of prior TB treatment episodes (SD)	2.5 (1.1)	3.5 (1.3)	0.08
Unfavorable treatment outcome of the last episode (%N)	41 (76)	3 (75)	0.69
Median time between MDR-TB diagnosis and treatment, days (minimum-maximum)	272 (26–888)	255 (193–317)	0.8
Mean percent of lung destruction (SD)	53 (23)	86 (11.7)	0.05
Median duration of intensive phase, months (IQR)	7 (6–8)	n/a	n/a
Median time to culture conversion, months (IQR)†	2 (1–3)	n/a	n/a

* Excludes 3 patients that defaulted.

† Excludes 8 patients for whom follow-up sputum culture results unavailable.

N/a =  not applicable.

Cavitary disease was present in 22 (36%) of the 48 patients for whom a pretreatment chest radiograph was available for analysis. Overall, patients had a significant cumulative improvement in the percent of damaged lung at the 6-month chest radiograph (HIV uninfected with mean improvement of 20% and estimated residual involvement of 37%, 95% CI 30–44%, p<0.001; and HIV infected with mean improvement of 14% and 32% residual, 95% CI 12–53%, p = 0.004). A significant increase was also observed in weight (HIV uninfected with mean gain of 5.9 kg and 6 month mean of 58.7 kg, 95% CI 55.3–62.1 kg, p<0.001; and HIV infected with mean gain of 6.2 kg and 6 month mean of 59.1 kg, 95% CI 46.2–72.0 kg, p = 0.009).

Pretreatment *M. tuberculosis* isolates were later recovered from only 28 (46%) patients for MIC testing to the second-line drugs within the MDR-TB treatment regimen. 15 patients with recovered isolates had culture converted ≥3 months and it was observed that 2 (13%) patients had isolates with high MIC to ofloxacin and kanamycin predictive of in vitro resistance and 6 (40%) had MICs predictive of resistance to ethionamide (Table 3). In contrast, all isolates had low MICs to PAS. None of the 4 patients that died had isolates recovered.

**Table 3:** Additional second-line susceptibility testing of recovered isolates.

Drug	MIC µg/ml (IQR)	Total, N = 28	Conversion <3 mo., N = 13	Conversion ≥3 mo., N = 15
		Resistant (%N)	Resistant (%N)	Resistant (%N)
Ofloxacin	0.5 (IQR 0.5–1.0)	3 (11)	1 (8)	2 (13)
Moxifloxacin	0.12 (IQR 0.06–0.25)	4 (14)	1 (8)	1 (7)
Amikacin	0.25 (IQR 0.25–0.5)	1 (4)	0	1 (7)
Kanamycin	1.2 (IQR 1.2–2.5)	3 (11)	1 (8)	2 (13)
Ethionamide	2.5 (IQR 1.2–5.0)	9 (32)	3 (23)	6 (40)
PAS	≤0.5 (IQR 0) (min ≤0.5-max 1.0)	0	0	0
Cycloserine*	8.0 (IQR 8.0–16.0)	n/a	n/a	n/a

Pyrazinamide susceptibility was not performed. PAS = para-aminosalicylic acid. Conversion =  time to sputum culture conversion to negative in months (mo). Resistance determined by minimum inhibitory concentration (MIC) analysis on MYCOTB Sensititre plates (TREK Diagnostics) with the following resistance breakpoints (ofloxacin 2.0 µg/ml; moxifloxacin 0.25 µg/ml; amikacin 1.0 µg/ml, kanamycin 5.0 µg/ml, ethionamide 5.0 µg/ml; PAS 2.0 µg/ml)[21;27].

* Resistance correlation with MIC in liquid media is not well established for cycloserine. One subject with culture conversion ≥3 months was resistant to both ofloxacin and kanamycin, considered extensively drug-resistant (XDR-TB).

## Discussion

This work describes the first cohort of patients treated for MDR-TB in Tanzania. Most were able to complete the intensive phase of inpatient therapy. Nearly all experienced medication related events, but few were severe or requiring of regimen alteration. While sputum culture conversion was delayed up to 8 months for some patients, the median duration fell within expected norms from other recent cohorts [7], [10].

There is a paucity of published data from MDR-TB treatment programs in Africa. However in comparison to the reports of integrated HIV/MDR-TB treatment programs from South Africa [7], [14], [15] we found a similarly high rate of sputum culture conversion among the subjects referred for MDR-TB treatment in Tanzania. Comprehensive programs from Lesotho have demonstrated that outcomes among HIV/MDR-TB patients can approach those of HIV uninfected patients [16] and this study provides further evidence of that possibility, albeit among a HIV infected cohort without profound immunosuppression. Despite a HIV prevalence of 14%, which is lower than the expected proportion among TB patients in Tanzania, all HIV infected subjects included in this study had CD4 counts >300 cells/µl at the time of MDR-TB treatment initiation and two-thirds were on ART prior to presentation. In addition, the vast majority of all patients had two or more prior episodes of TB, thus it is suspected that the entire study population was enriched for those with drug resistance acquired on therapy that were relatively healthy enough to survive the prolonged referral process prior to treatment. This has not been the case from South Africa where transmitted drug-resistant strains are thought to fuel the epidemic [15]. Yet in general, studies from southern Africa acknowledge a similar survival bias of subjects referred for treatment initiation [7], and not until widespread efforts at intensified case finding with rapid diagnostics for drug-resistance are implemented can we assess the true efficacy of the MDR treatment program in a given region.

We remain encouraged that the cumulative increase in weight and radiographic resolution in our cohort are further suggestive of ultimate treatment success [17]. Indeed, in comparison to those completing therapy, patients that died had a trend toward more extensive lung disease. Pretreatment isolates were not recovered from the patients that died for second-line susceptibility testing, although it is of additional concern that resistance to key medications such as fluoroquinolones or aminoglycosides may have contributed to the unfavorable clinical response. We chose to perform MIC testing because the commercial 96-well MIC plate was relatively straightforward to deploy compared to the cumbersome agar proportion methodology. Of the second-line MIC testing performed on the available isolates, resistance to ofloxacin and kanamycin was rare but not absent and a worrisome trend of the highest MICs was observed in patients with delayed culture conversion. As has been observed, MICs were lower for moxifloxacin compared to ofloxacin, as well as for amikacin compared to kanamycin [18]. We have previously noted low plasma concentrations of anti-TB medications at KNTH, therefore it stands to reason that optimization of pharmacokinetics within a class may further improve outcome [19]. In addition, of the oral second-line agents, PAS appeared more reliably susceptible than ethionamide. While the use of MICs in lieu of the standard qualitative susceptibility methods requires further study, in our view there exists appeal in examining the use of MICs for regimen individualization.

Other MDR-TB cohorts have reported a larger number of patients stopping medication due to severe adverse events, described in up to 55% of all subjects and even death related to hepatic failure [20]–[23]. Our rates were lower, which we suspect was biased by survival to referral in a population with considerable prior exposure and tolerance to first-line anti-tuberculous medications including pyrazinamide. While our analysis was restricted to the inpatient intensive phase, common side effects included arthralgia and headache, which may be difficult to ascribe to medication alone, though certainly pyrazinamide may cause arthralgia in the setting of urate arthropathy [24]. 20% of patients experienced peripheral neuropathy likely due to ethionamide, cycloserine or the additive combination. A more aggressive strategy of pyridoxine supplementation may therefore be of benefit.

Fortunately, aminoglycoside related nephrotoxicity and ototoxicity were uncommon despite monthly monitoring. Notably however, cycloserine related psychosis was morbid when observed, necessitating temporary drug discontinuation and antipsychotic treatment. Patients with this complication were HIV negative and therefore not on efavirenz, which has been previously suggested as additive to psychiatric side effects [25]. Terizidone, a derivative of cycloserine, may be less toxic and is a preferred alternative in similar treatment programs [26]. Medication toxicity notwithstanding, a significant proportion of subjects had treatment change due to periodic stock shortage. Reliable procurement is a realistic obstacle that must be considered for similar resource-limited settings planning MDR-TB treatment and we recommend as becoming integral to staff and facility training.

There are several limitations to this study, largely implicit to retrospective analysis. Further detail about medication change or interventions to ameliorate side effects or assure adherence were unavailable. As discussed, the relative homogeneity of the population may also limit generalizability. The favorable interim outcomes from this initial cohort may be dampened as rapid diagnostics for MDR-TB are more widely available in Tanzania, which will likely identify sicker patients that previously would not have survived to referral. Lastly, lack of routine second-line susceptibility testing did not allow for determination of flouroquinolone or injectable agent resistance in all patients and hence the proportion that may have harbored extensively drug-resistant TB and the contribution of such resistance to treatment outcome is unknown.

Of further cautionary note, we found non-tuberculous mycobacteria typically resistant to certain TB medication that may otherwise have been misclassified as MDR-TB without confirmatory speciation. Additionally, the isoniazid and rifampicin monoresistance detected would greatly alter the treatment regimen. Thus, this study reinforces the need for laboratory capacity beyond testing for *rpo*B mutation to exist alongside any new MDR-TB program.

In summary, the majority of patients within the first cohort treated for MDR-TB in Tanzania completed an intensive phase of inpatient therapy without serious adverse events. Patients had a prior history of TB treatment, likely acquired drug-resistance, did not have significant immunosuppression and were relatively healthy enough to have survived a prolonged referral process. Countries with similar infrastructure that are entertaining start of a centralized MDR-TB program might anticipate similar findings, yet further follow-up of the long-term outcomes remains. Additional setting-specific study can determine the clinical impact of quantitative susceptibility testing for medications within the MDR-TB regimen, and the feasibility of alternatives to prolonged hospitalization. As efforts are underway to scale-up MDR-TB diagnosis for other locations in Tanzania, such studies are urgently needed.
